# Allelic variation in CYP3A4 and PLB1 drives feed efficiency and immunometabolic resilience in beef cattle

**DOI:** 10.1186/s12864-025-12412-0

**Published:** 2025-12-18

**Authors:** Olanrewaju B. Morenikeji, Modoluwamu Idowu, Godstime Taiwo, Luke M. Gratz, Blessing Olabosoye, Raven E. King, Nadiya D. Andrews, Fatimatou Saccoh, Anastasia Grytsay, Maneiqua C. Marshall, Ibukun M. Ogunade

**Affiliations:** 1 Department of Biology, Virginia State University, Petersburgh, VA 23806 USA; 2 Division of Biological and Health Sciences, University of Pittsburgh at Bradford, Bradford, PA 16701 USA; 3 Division of Animal and Nutritional Science, West Virginia University, Morgantown, WV 26505 USA; 4 School of Agriculture, Tennessee Technological University, Cookeville, TN 38501 USA; 5 Department of Animal Science and Range Sciences, New Mexico State University, Las Cruces, NM 88003 USA; 6 Department of Animal Science, Iowa State University, Pammel Drive, Ames, IA USA

**Keywords:** Immunometabolism, RFI, Gene polymorphisms, Haplotype, Immunocompetence

## Abstract

Immunometabolic traits are increasingly recognized as key determinants of feed efficiency and resilience in beef cattle. This study aimed to identify functional genetic markers associated with these traits by integrating SNP genotyping and gene expression profiling. We evaluated genetic markers for feed efficiency and immunocompetence in 102 crossbred steers (217 ± 8.2 kg) fed a high-forage total mixed ration for 63 days, using GrowSafe8000 intake nodes to calculate residual feed intake (RFI). From the 20 most efficient (low-RFI) and 20 least efficient (high-RFI) animals, we genotyped three metabolic loci (CYP3A4 rs438103222, PLB1 rs456635825, CRAT rs876019788) and profiled blood mRNA levels of these, plus eight innate/adaptive immune genes. Logistic regression revealed that CYP3A4 and PLB1 polymorphisms, but not CRAT, were strongly associated with initial and final body weight, average daily gain, and feed intake: CYP3A4 A/A and PLB1 A-allele carriers achieved superior growth on reduced feed. Haplotype reconstruction across the three loci defined eight multi-SNP combinations, with the C-A-A haplotype enriched in low-RFI steers and combinations harboring CYP3A4 A and PLB1 A alleles linked to low RFI. Intriguingly, these favorable genotypes also overlapped with up-regulation of immune sensors and effectors (e.g., CD14, TLR4, TNF-α), indicating a coordinated metabolic–immune adaptation in efficient cattle. Collectively, our results validate CYP3A4 and PLB1 as high-impact quantitative trait nucleotides for marker-assisted selection aimed at simultaneously improving feed efficiency and immune resilience in beef production.

## Introduction

Optimizing feed efficiency is paramount for sustainable beef production, and residual feed intake (RFI), the deviation between observed and predicted feed consumption for a given growth rate has emerged as a robust metric for selecting cattle with superior nutrient utilization and lower environmental footprint [1, 2]. In ruminants, effective feed efficiency hinges on the integration of complex rumen fermentation dynamics with systemic metabolic pathways tailored to a herbivorous diet [3, 4]. Importantly, energetic imbalances and accumulation of toxic metabolites can compromise immune homeostasis, linking nutritional status directly to host defense mechanisms and disease resilience [5, 6]. Building on prior evidence linking metabolic genes to feed efficiency and immune modulation [7, 8], we selected CYP3A4, PLB1, and CRAT for targeted analysis. These genes encode enzymes central to detoxification (CYP3A4), lipid remodeling (PLB1), and mitochondrial energy metabolism (CRAT), each with established roles in nutrient partitioning and immune cell activation. Prior cattle QTL and GWAS studies have linked genomic regions associated with feed intake, fat deposition, liver function, and energy balance [9–11], though these studies did not explicitly name CYP3A4, PLB1, or CRAT. This reinforces the novelty of our study and their relevance to immunometabolic regulation in beef cattle.

At the molecular level, genetic variation in key metabolic enzymes can profoundly influence both energy partitioning and immunometabolism. CYP3A4, a cytochrome P450 heme enzyme, catalyzes phase I oxidation of a broad spectrum of endogenous and exogenous substrates including dietary phytochemicals, mycotoxins, steroid hormones, and xenobiotics, and its activity depends on precise substrate–heme interactions mediated by critical amino acid residues [12–14]. A non-synonymous C >A SNP resulting in glycine-to-cysteine substitution can perturb enzyme conformation, reducing detoxification capacity and leading to toxicant accumulation that impairs leukocyte function [15, 16].

Carnitine O-acetyltransferase (CRAT) orchestrates mitochondrial acetyl‐CoA/carnitine interconversion, a pivotal node for fatty acid β-oxidation and ATP generation that fuels proliferating immune cells [17, 18]. A valine‐to‐phenylalanine substitution in CRAT may alter substrate channeling or enzyme stability, with potential downstream effects on energy‐dependent immune responses. Phospholipase B1 (PLB1) regulates membrane lipid remodeling and generates bioactive lysophospholipids essential for cell signaling, inflammation, and antigen presentation; a G >A SNP converting leucine to phenylalanine could disrupt PLB1’s membrane association or catalytic efficiency, attenuating both lipid homeostasis and immunomodulatory lipid mediator synthesis [19, 20].

Comparative genomic analyses reveal that CYP3A4 and PLB1 are conserved across mammalian species, with functional orthologs in humans, pigs, and rodents contributing to detoxification and lipid signaling pathways [21]. CYP3A4 expression is modulated by nuclear receptors such as PXR and CAR, which respond to dietary and xenobiotic stimuli, while PLB1 activity is influenced by phospholipid turnover and inflammatory cues [14]. CRAT, though less variable in expression, plays a pivotal role in mitochondrial acetyl-CoA flux and is regulated by nutrient-sensing pathways including AMPK and PPARα [22]. These regulatory networks suggest that feed efficiency traits may emerge from coordinated transcriptional responses to metabolic stress, with SNPs in these genes acting as modulators of enzymatic activity and immune readiness.

Despite growing evidence for immunometabolic crosstalk in livestock, few studies have integrated performance phenotypes, blood transcriptomics, and high-resolution haplotype mapping to pinpoint functional QTNs for both feed efficiency and immune competence. To address this gap, our study aimed to identify functional SNPs in CYP3A4, CRAT, and PLB1 among crossbred steers divergently selected for RFI, and to evaluate their associations with metabolic and immune gene expression profiles. We interrogated SNP variability in *CYP3A4* (*rs438103222*), *CRAT* (*rs876019788*), and *PLB1* (*rs456635825*) among crossbred steers divergently selected for RFI, profiling metabolic and immune gene expression in blood. By combining logistic regression, cis‐/trans‐eQTL analyses, and linkage‐haplotype reconstruction, we elucidate how these polymorphisms coordinate detoxification, lipid metabolism, and innate/adaptive immunity, laying the groundwork for marker‐assisted selection strategies that simultaneously enhance productive efficiency and disease resilience in beef cattle [23, 24].

## Materials and methods

### Experimental procedures and data collection

All animal protocols were reviewed and approved by the West Virginia University Animal Care and Use Committee (Protocol No. 2204052569.1). One hundred and two crossbred Angus × Hereford growing steers (initial BW = 217 ± 8.2 kg), originating from a uniform structured crossbreeding program in which breed proportions were consistent across all individuals, were managed together at West Virginia University research farm prior to the study. Steers were housed in a confinement drylot and provided *ad libitum* access to a high-forage total mixed ration and fresh water. Individual feed intake and body weight were recorded continuously over a 63-day test using GrowSafe^®^ intake nodes and automated weighing systems. Daily dry matter intake (DMI) was calculated from the real‐time intake records, and residual feed intake (RFI) was computed as the difference between observed DMI and predicted DMI based on maintenance and weight‐gain requirements as described by [25]. At the end of the trial (d 63), whole blood was drawn from each steer via jugular venipuncture before the morning meal into sodium‐heparin tubes. An aliquot of each sample was immediately transferred into Qiagen RNAprotect Blood Tubes, mixed according to the manufacturer’s instructions for mRNA stabilization, and stored at − 80 °C until processing. Thereafter, steers were ranked by RFI, and 40 animals were chosen for further molecular analyses: the 20 with the lowest RFI (most feed‐efficient) and the 20 with the highest RFI (least feed‐efficient). Daily body weights and actual DMI values for these 40 steers formed the basis for all subsequent performance and expression studies.

### RNA Isolation, cDNA synthesis and gene expression

Genomic DNA and total RNA were co-isolated from the same blood specimens from the 40 steers using Qiagen RNeasy Kits. DNA yield and purity were quantified on a NanoDrop 2000 (A260/A280 ratio), and integrity was confirmed by 1% agarose gel electrophoresis. Total RNA concentration was likewise measured on the NanoDrop, and integrity was assessed on an Agilent Bioanalyzer; only samples with RIN > 8.0 and A260/A280 ratios between 1.8 and 2.0 were advanced to cDNA synthesis using the Qiagen RT² First Strand Kit. Gene‐specific primers (Table 1) were designed for three metabolic targets (CYP3A4, CRAT, PLB1) and eight immune markers (CD14, TLR4, TNF-α, CEBPB, ITGAM, IRF1, TLR2, RHOA, NANS). Quantitative PCR was performed on a Bio-Rad CFX Opus Real-Time System using initial denaturation at 95 °C for 10 min, followed by 40 cycles of 95 °C for 15 s and 60 °C for 1 min. Relative transcript abundance in low-RFI versus high-RFI groups was calculated by the 2⁻ΔΔCt method in Bio-Rad Maestro, with β-actin and GAPDH serving as endogenous controls.

**Table 1:** Gene primers for selected metabolic and immune gene expression analysis

Gene Name	Primer Sequence	Product Length	Tm	GC%
CEBPB Forward	AGAAGACGGTGGACAAGCAC	98	60.25	55.00
CEBPB Reverse	GTTGCGCATCTTGGCCTTG		60.44	57.89
IRF1 Forward	ATCTTGTGGGGTGAAGCTGG	108	59.96	55.00
IRF1 Reverse	CTCCAAGGGGAAAGCTGGAG		60.03	60.00
RHOA Forward	GATGTCCAACCCACCTGACC	92	60.32	60.00
RHOA Reverse	AATTAGCGCCTGGTGTGTCA		59.96	50.00
NANS Forward	GCTCTTTCCTGACATCCCCAT	105	59.79	52.38
NANS Reverse	GTTATGTGACGCTCCAAGACC		59.00	52.38
CD-14 Forward	GACACCAACCCGAAGCAGTA	95	59.97	55.00
CD-14 Reverse	ACCAGAAGCTGAGCAGGAAC		59.96	55.00
TLR-2 Forward	CTTCCTGTTGCTCCTGCTCA	107	59.96	55.00
TLR-2 Reverse	CCTTCCTGGGCTTCCTCTTG		60.03	60.00
TLR-4 Forward	GGTGGAGCTCTATCGCCTTC	120	59.97	60.00
TLR-4 Reverse	CTCTGGGGTTTACCAGCCAG		60.04	60.00
TNF-A Forward	GGACACCCAGAATGTGAGGG	102	60.04	60.00
TNF-A Reverse	GGAGAGTTGAAGTCCACGCA		59.97	55.00
ITGAM Forward	AAGTTGAGGCGACGATGGAG	101	60.11	55.00
ITGAM Reverse	TTTCACCTGCCCAGCAATCT		59.89	50.00
CRAT Forward	ATTCCTCCTCGCTCACGATG	105	59.61	55
CRAT Reverse	TTAAGGCACACCAGGACTCG		59.58	55
PLBI Forward	TAGAAGAAGGGCTGGAAGACG	101	59.17	52.38
PLBI Reverse	TGACGGTACTCCTTTCTTCAGG		59.44	50
CYP3A4 Forward	ACCTGGAAGTCCAGATGTTCA	115	58.66	47.62
CYP3A4 Reverse	AGGAAATACCCATGTCCCTACC		58.95	50

### TaqMan SNP genotyping assay and allele discrimination

SNP genotyping analysis was performed on SNPs of *CYP3A4* (*rs438103222*), *PLB1* (*rs456635825*), and *CRAT* (*rs876019788*) using custom designed TaqMan SNP genotyping assays and ordered from (Thermo Fisher Scientific, Waltham MA). Each assay was conducted in a 10 µl reaction volume containing 1 µl of 20X TaqMan SNP genotyping assay, 5 µl of 2× TaqMan Mastermix, 1 µl of 20 ng genomic DNA, and 3 µl of nuclease-free water. Real-time PCR was performed on a Bio-Rad CFX Opus machine (Bio-Rad, Hercules, CA) using the following conditions: 90 °C for 10 min, followed by 30 cycles of 90 °C for 30 s, 56 °C for 30 s, and 72 °C for 50 s. A final extension step was performed at 72 °C for 5 min. Melt curve analysis was conducted to confirm assay specificity, with temperatures ranging from 65 °C to 95 °C in 0.5 °C increments. Continuous fluorescent measurements were taken during this process. Allele calls and discriminations were generated using the Bio-Rad Maestro software (Bio-Rad, Hercules, CA). SNP selection was guided by functional annotation and predicted impact on protein activity. All three variants are located within exonic regions and result in amino acid substitutions: glycine-to-cysteine in CYP3A4, leucine-to-phenylalanine in PLB1, and valine-to-phenylalanine in CRAT. These substitutions occur in conserved domains implicated in substrate binding or catalytic efficiency.

### SNP analysis and association testing /statistical analysis

Allelic and genotypic frequencies for SNPs of CYP3A4 (rs438103222), PLB1 (rs456635825), and CRAT (rs876019788) were calculated using SNPstats [26]. Deviations from Hardy-Weinberg equilibrium was assessed, with SNPs rejected at a p-value threshold of 0.05. Association analysis between these SNPs, RFI status, performance characteristics and gene expressions data were conducted using Fisher’s exact test. Allelic and genotypic frequencies were compared between high and low RFI animal groups, as previously described [27].

Genotype–phenotype associations were evaluated under codominant, dominant, and recessive inheritance models using logistic regression. Model selection was based on Akaike Information Criterion (AIC) to identify the best-fitting inheritance pattern. To account for multiple comparisons across genotypes and traits, Benjamini-Hochberg false discovery rate (FDR) correction was applied to control for type I error inflation. Gene expression differences across genotypes and RFI groups were analyzed using one-way ANOVA followed by Tukey’s post hoc test for multiple group comparisons. Fold-change values were calculated using the 2⁻ΔΔCt method, and statistical significance was determined using adjusted p-values (FDR < 0.05). We further evaluated linkage disequilibrium among these loci to assess potential epistatic interactions and coordinated expression patterns. All statistical analyses and graphs were performed using GraphPad Prism 9.0 and R version 4.2.1, with packages including stats, multcomp, and p.adjust. These methods ensure robust interpretation of genotype–phenotype and genotype–expression relationships across the study. Additionally, haplotype analysis was performed for the three SNPs, excluding animals heterozygous at multiple loci.

## Results

### Growth performance of the beef steers

Initial BW did not differ between low- and high-RFI cattle (211.5 vs. 215.2 kg; *P* = 0.78; Table 2). Similarly, final BW was not affected by RFI classification (304.8 vs. 307.8 kg for low- and high-RFI, respectively; *P* = 0.54). Total BW gain was comparable between groups (93.3 vs. 92.6 kg; *P* = 0.89), and no differences were detected in ADWG (1.48 vs. 1.51 kg/d; *P* = 0.81). Dry matter intake was greater (*P* = 0.04) in high-RFI cattle (8.41 kg/d) compared with low-RFI cattle (7.06 kg/d). As expected, RFI values differed between the two RFI classes (− 1.95 vs. 1.56 kg/d; *P* = 0.01).

**Table 2:** Growth performance of the RFI-divergent beef steers

	Low-RFI	High-RFI	SEM	*P*-values
Initial Weight, Kg	211.5	215.2	7.32	0.78
Final Weight, Kg	304.8	307.8	2.12	0.54
TBWG, Kg	93.3	92.6	8.14	0.89
ADWG, Kg/d	1.48	1.51	0.06	0.81
DMI, Kg/d	7.06	8.41	0.58	0.04
RFI, Kg/d	-1.95	1.56	0.64	0.01

*TBWG* total body weight gain, *ADWG* average daily weight gain, *DMI* dry matter intake, *RFI* residual feed intake

### Metabolic gene SNP-driven variations as determinants of cattle performance

We evaluated the impact of gene polymorphisms on performance traits by conducting logistic regression analyses to assess genotypic frequency distributions, with significance levels determined for each SNP in *CYP3A4* (*rs438103222*), *PLB1* (*rs456635825*), and *CRAT* (*rs876019788*). Various genetic models (codominant, dominant, and recessive) were applied to explore associations with initial weight (IW), final weight (FW), average daily weight gain (ADWG), total weight gain (TWG), and average daily feed intake (ADFI) (Tables 3, 4 and 5).

Our results show significant effects of polymorphisms in *CYP3A4* and *PLB1*, but not in *CRAT*. Table 3 illustrates that the mutant genotype (AA) of *CYP3A4* is associated with the highest IW (223.33 kg) and FW (275.3 kg) compared to wild-type and heterozygous genotypes. The wild-type genotype (CC) showed the highest ADFI (8.67 kg). Heterozygotes (CA) maintained intermediate values across IW (210 kg), FW (255 kg), ADWG (1.3 kg), and TWG (45 kg), except for a lower ADFI (4.93 kg). Under the dominant model, AA animals achieved comparable TWG and ADWG to CC despite consuming only half as much feed, suggesting an enhanced feed conversion efficiency.

At PLB1 rs456635825, shown in Table 4, the minor A allele exerts a dosage-dependent anabolic effect on bovine growth and feed efficiency: under an additive inheritance model, A/A steers displayed significantly higher initial body weight (225.5 kg vs. 213.4 kg in G/A; *p* < 0.05), greater final weight (278.2 kg vs. 267.5 kg in G/A), elevated average daily weight gain (1.51 kg/d vs. 1.12 kg/d in G/G; *p* < 0.01), augmented total weight gain (52.7 kg vs. 30.3 kg in G/G; *p* < 0.001) and reduced average daily feed intake (5.20 kg/d vs. 8.13 kg/d in G/G; *p* < 0.05), with heterozygous G/A phenotypes intermediate to homozygotes. A recessive model (A‐carriers vs. G/G) further confirmed the A allele’s effect, carriers exhibited superior growth kinetics (+ 0.41 kg/d ADWG; *p* < 0.001), increased cumulative gain (+ 23.3 kg TWG; *p* < 0.001) and lower feed intake (− 2.91 kg/d ADFI; *p* < 0.01). Although the dominant contrast (A/A vs. G‐carriers) trended similarly, statistical power was constrained by the small G/G cohort. These data identify the A allele at PLB1 rs456635825 as a potent quantitative trait nucleotide for marker‐assisted selection aimed at enhancing bovine production metrics.

**Table 3:** CYP3A4 genotypes and their effects on growth and feed intake traits

Polymorphism	Genotype	IW	FW	ADWG	TWG	ADFIM
CYP3A4 (rs438103222)	C/C	218.25 (5.15)	271.92 (5.44)	1.53 (0.04)	52.4 (2.29)	8.67 (3.32)
	C/A	210.38(6.06)	255.83 (7.74)	1.3 (0.1)	45.45(3.42)	4.93 (0.13)
	A/A	223.33(13.67)	275.3 (13.85)	1.48 (0.07)	51.97 (2.45)	4.73 (0.19)
	Dominant					
	C/C	218.25(5.15)	271.92 (5.44)	1.53 (0.04)	52.4 (2.29)	8.67 (3.32)
	C/A - A/A	216.86(7.39)	265.57 (8.11)	1.39 (0.06)	48.71 (2.23)	4.83 (0.11)
	Recessive					
	C/C - C/A	216.86(4.37)	269.08 (4.76)	1.49(0.04)	51.17 (2.02)	8.01 (2.73)
	A/A	223.33(13.67)	275.3 (13.85)	1.48 (0.07)	51.97 (2.45)	4.73 (0.19)

Asterisks denote statistical significance: *p* < 0.05 (*), *p* < 0.01 (**); ns or blank = not significant

*IW* Initial Weight, *FW* Final Weight, *ADWG* Average Daily Weight Gain, *TWG* Total Weight Gain, *ADFI* Average Daily Feed Intake

**Table 4:** PLB1 genotypes and their effects on growth and feed intake traits

Model	Genotype	IW	FW	ADWG	TWG	ADFI
PLB1 (rs456635825)	A/A	225.49(8.36) *	278.18 (8.26)	278.18 (8.26)	52.69 (1.43) ***	5.2 (0.19) *
	G/A	213.39(4.99)	267.48 (5.56)	1.55 (0.05)	54.09 (1.68)	5.24 (0.14)
	G/G	221.48(15.92)	267.48 (5.56)	1.12 (0.08)	30.31 (8.88)	8.13 (3.33)
	Dominant					
	A/A	225.49(8.36)	278.18(8.26)	1.51 (0.04)	52.69 (1.43)	5.2 (0.19)
	G/A - G/G	214.55(4.74)	266.51 (5.3)	1.48 (0.05)	50.69 (2.42)	8.51 (3.32)
	Recessive					
	A/A - G/A	217.42(4.38)	271.05(4.63)	1.53 (0.03) ***	53.62(1.21) ***	5.22 (0.11) **
	G/G	221.48(15.92)	260.68 (18.3)	1.12 (0.08)	30.31 (8.88)	8.13 (3.33)

Asterisks denote statistical significance: *p* < 0.05 (*), *p* < 0.01 (**); ns or blank = not significant

*IW* Initial Weight, *FW* Final Weight, *ADWG* Average Daily Weight Gain, *TWG* Total Weight Gain, *ADFI* Average Daily Feed Intake

For CRAT (rs876019788), the genotypic effects are presented in Table 5. The valine-to-phenylalanine allelic substitution exerts no consistent modulatory effect on bovine growth or feed utilization. Under an additive model, A/A homozygotes (IW 213.8 ± 4.9 kg; FW 267.1 ± 5.4 kg; ADWG 1.52 ± 0.05 kg/d; TWG 53.3 ± 1.8 kg; ADFI 5.20 ± 0.12 kg/d) and C/C homozygotes (IW 222.6 ± 7.2 kg; FW 274.0 ± 7.6 kg; ADWG 1.47 ± 0.04 kg/d; TWG 51.4 ± 1.4 kg; ADFI 5.19 ± 0.19 kg/d) display virtually overlapping weight-gain kinetics and feed intake, while A/C heterozygotes exhibit aberrantly high intake variance (ADFI 23.72 ± 18.61 kg/d) without proportional gain enhancement. Higher ADFI observed for the A/C genotype should be interpreted with caution. The relatively small A/C group and sampling variability possibly introduced outliers that contributed to the observed difference. Dominant (A-carriers vs. C/C) and recessive (A/A vs. C-carriers) contrasts further confirm negligible genotype-phenotype associations in ADWG, TWG, and ADFI. Collectively, rs876019788 does not qualify as a quantitative trait nucleotide for feed efficiency or growth metrics in beef cattle.

**Table 5:** CRAT genotypes and their effects on growth and feed intake traits

Model	Genotype	IW	FW	ADWG	TWG	ADFI
CRAT (rs876019788)	A/A	213.82(4.88)	267.1(5.41)	1.52 (0.05)	53.28 (1.79)	5.2 (0.12)
	A/C	231.82(17.33)	279.36 (18.41)	1.36 (0.08)	40.43 (9.15)	23.72 (18.61)
	C/C	222.61(7.19)	273.98 (7.57)	1.47 (0.04)	51.36 (1.43)	5.19 (0.19)
	Dominant					
	A/A	213.82(4.88)	267.1(5.41)	1.52 (0.05)	53.28 (1.79)	5.2 (0.12)
	A/C - C/C	226.15(7.67)	276.05 (8.04)	1.43 (0.04)	47.16 (3.72)	12.32 (7.15)
	Recessive					
	A/A - A/C	216.63(4.93)	269.02 (5.31)	1.5 (0.05)	51.27 (2.16)	8.1 (2.91)
	C/C	222.61(7.19)	273.98 (7.57)	1.47 (0.04)	51.36 (1.43)	5.19 (0.19)

Asterisks denote statistical significance: *p* < 0.05 (*), ns or blank = not significant

*IW* Initial Weight, *FW* Final Weight, *ADWG* Average Daily Weight Gain, *TWG* Total Weight Gain, *ADFI* Average Daily Feed Intake

Across both high- and low-RFI cohorts from Table 6, CYP3A4 (rs438103222) exhibits a pronounced heterozygote disadvantage: C/A steers show significantly reduced average daily weight gain (ADWG) and total weight gain (TWG) relative to C/C and A/A homozygotes (e.g., high-RFI C/A: 1.38 ± 0.07 kg/d vs. C/C: 1.56 ± 0.06 kg/d and A/A: 1.50 ± 0.08; low-RFI C/A: 0.91 kg/d vs. C/C: 1.52 ± 0.06 and A/A: 1.46 ± 0.19), implicating disrupted CYP3A4 catalytic efficiency when glycine/cysteine residues are paired. In stark contrast, PLB1 (rs456635825) minor-allele homozygotes (G/G) demonstrate a recessive growth penalty with ADWG reducing to 1.15 ± 0.01 kg/d and aberrant ADFI (51.46 kg/d) in high-RFI steers versus ~ 1.53 kg/d ADWG and ~ 5.2 kg/d ADFI in A-allele carriers signifying deleterious effects of the leucine-to-phenylalanine substitution on phospholipase B1 function. Conversely, the CRAT (rs876019788) valine-to-phenylalanine polymorphism fails to produce genotype-dependent differences in initial or final weight, ADWG, TWG, or ADFI within either RFI group, indicating its negligible role in feed-efficiency phenotypes. These patterns position CYP3A4 and PLB1 as key quantitative trait nucleotides for marker-assisted selection to optimize bovine growth and feed conversion relative to RFI status, while CRAT lacks actionable significance.

**Table 6:** Combined genotypic effects of CYP3A4, PLB1, and CRAT on performance traits by RFI group

RFI	Gene	Genotype	IW	FW	ADWG	TWG	ADFI
High	CYP3A4	C/C	231.40(8.92)	285.99 (9.5)	1.56 (0.06)	51.35(4.88)	13.99 (8.42)
		C/A	214.18(5.78)	262.36 (5.08)	1.38 (0.07)	48.18 (2.53)	4.94 (0.16)
		A/A	216.82(16.57)	269.2 (18.73)	1.5 (0.08)	52.39 (2.75)	4.71 (0.3)
Low		C/C	209.73(5.47)	262.81 (5.69) *	1.52 (0.06)	53.07(2.21)	5.22 (0.17)
		C/A	191.36(N/A)	223.18 (---) *	0.91 (---) *	31.82 (---)	4.87 (---)
		A/A	236.36(30.00)	287.5 (23.41)	1.46 (0.19)	51.14 (6.59)	4.77 (0.03)
High	PLB1	A/A	225.34(11.31)	277.84 (11.82)	1.5 (0.04)	52.5 (1.38) / 0.00	5.07 (0.25)
		G/A	225.27(8.57)	280.32 (9.36)	1.57 (0.06)	55.05 (1.99)	5.4 (0.2)
		G/G	214.09(2.27)	254.32 (2.05)	1.15 (0.01) *	22.44 (17.56) *	51.46 (46.67) *
		A/A	225.80(13.12)	278.86 (9.9)	1.52 (0.11)	53.07 (3.69)	5.47 (0.27)
		G/A	204.90(5.09)	258.31 (5.89)	1.53 (0.07)	53.41 (2.55)	5.12 (0.19)
		G/G	228.86(37.50)	267.05 (43.86)	1.09 (0.18) *	38.18 (6.36) *	4.8 (0.07)
High	CRAT	A/A	222.60(7.22)	277.23 (8.13)	1.56 (0.06)	54.63 (2.15)	5.31 (0.16)
		A/C	227.84(21.78)	276.36 (23.45)	1.39 (0.1)	39.63(11.77) *	28.22 (23.31) *
		C/C	224.73(11.16)	275.91 (12.23)	1.46 (0.05)	51.18 (1.72)	5.23 (0.31)
Low		A/A	207.78(6.32)	260.14 (6.89)	1.5 (0.08)	52.36 (2.67)	5.13 (0.18)
		A/C	247.73(N/A)	291.36 (---)	1.25 (---)	43.64 (---)	5.71 (---)
		C/C	219.09(7.62)	270.76 (5.36)	1.48 (0.09)	51.67 (3.03)	5.13 (0.17)

Asterisks denote statistical significance: *p* < 0.05 (*), ns or blank = not significant

*IW* Initial Weight, *FW* Final Weight, *ADWG* Average Daily Weight Gain, *TWG* Total Weight Gain, *ADFI* Average Daily Feed Intake, *RFI* Residual Feed Intake, *Low RFI* feed-efficient animals, *High RFI* feed-inefficient animals

## Differential metabolic transcriptome profiles in low- vs. high-RFI cattle

**Fig. 1:**
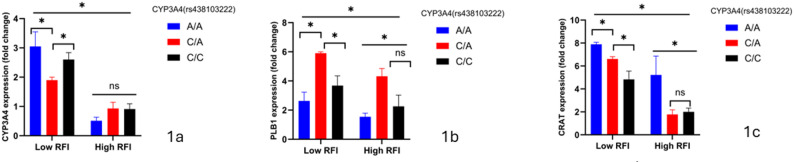
**a**-**c** CYP3A4 genotypes and relative expression of metabolic genes. Asterisks denote statistical significance: *p* < 0.05 (*), ns = not significant.Low RFI = feed-efficient animals; High RFI = feed-inefficient animals

**Fig. 2:**
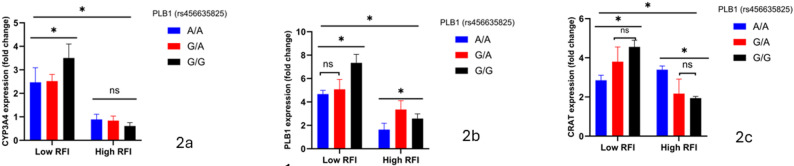
**a**-**c** PLB1 genotypes and relative expression of metabolic genes. Asterisks denote statistical significance: *p* < 0.05 (*), ns = not significant.Low RFI = feed-efficient animals; High RFI = feed-inefficient animals

**Fig. 3:**
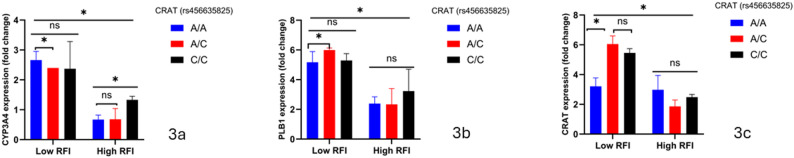
**a**-**c** CRAT genotypes and relative expression of metabolic genes. Asterisks denote statistical significance: *p* < 0.05 (*), ns = not significant.Low RFI = feed-efficient animals; High RFI = feed-inefficient animals

Across Figs. 1 and 3, blood-based cis‐eQTL mapping under divergent RFI phenotypes consistently demonstrates that the A allele at both CYP3A4 (rs438103222) and PLB1 (rs456635825) drives robust, allele-dosage–dependent up-regulation of its own transcript in efficient (low-RFI) cattle. Specifically, in low-RFI steers, CYP3A4 A/A homozygotes exhibit a 2.5-fold increase over C/C and a 1.8-fold rise relative to C/A (Fig. 1a and b; *p* < 0.05), while PLB1 A/A animals show a 2.1-fold augmentation versus G/G and intermediate G/A expression (Fig. 2a and b; *p* < 0.05). Importantly, these allele-specific expression differences are abrogated in high-RFI cohorts, underscoring a feed-efficiency–dependent enhancer effect. In contrast, CRAT (rs876019788) transcripts remain invariant across A/A, A/C, and C/C genotypes in both RFI groups (Fig. 3a and c; ns), indicating that its valine-to-phenylalanine substitution does not perturb cis-regulatory control.

Figure 2 expands this regulatory landscape by revealing PLB1’s A allele as a bifunctional quantitative trait nucleotide: beyond its cis-eQTL effect, low-RFI A/A and G/A steers also display significant trans-activation of CYP3A4 (Fig. 2a; *p* < 0.05) and CRAT (Fig. 2c; *p* < 0.05) transcripts, linking augmented phospholipase B1–mediated lipid remodeling to broader immunometabolic adaptation. High-RFI animals, however, lack this trans-regulatory cross-talk, highlighting a genotype×environment interaction that reinforces feed-efficiency endophenotypes. These data suggest CYP3A4 and PLB1 as important quantitative trait nucleotides whose allele-specific cis- and trans-regulatory activities drive key immunometabolic networks in beef cattle, while positioning CRAT as a distal regulatory hub subject to post-transcriptional modulation.

**Fig. 4:**
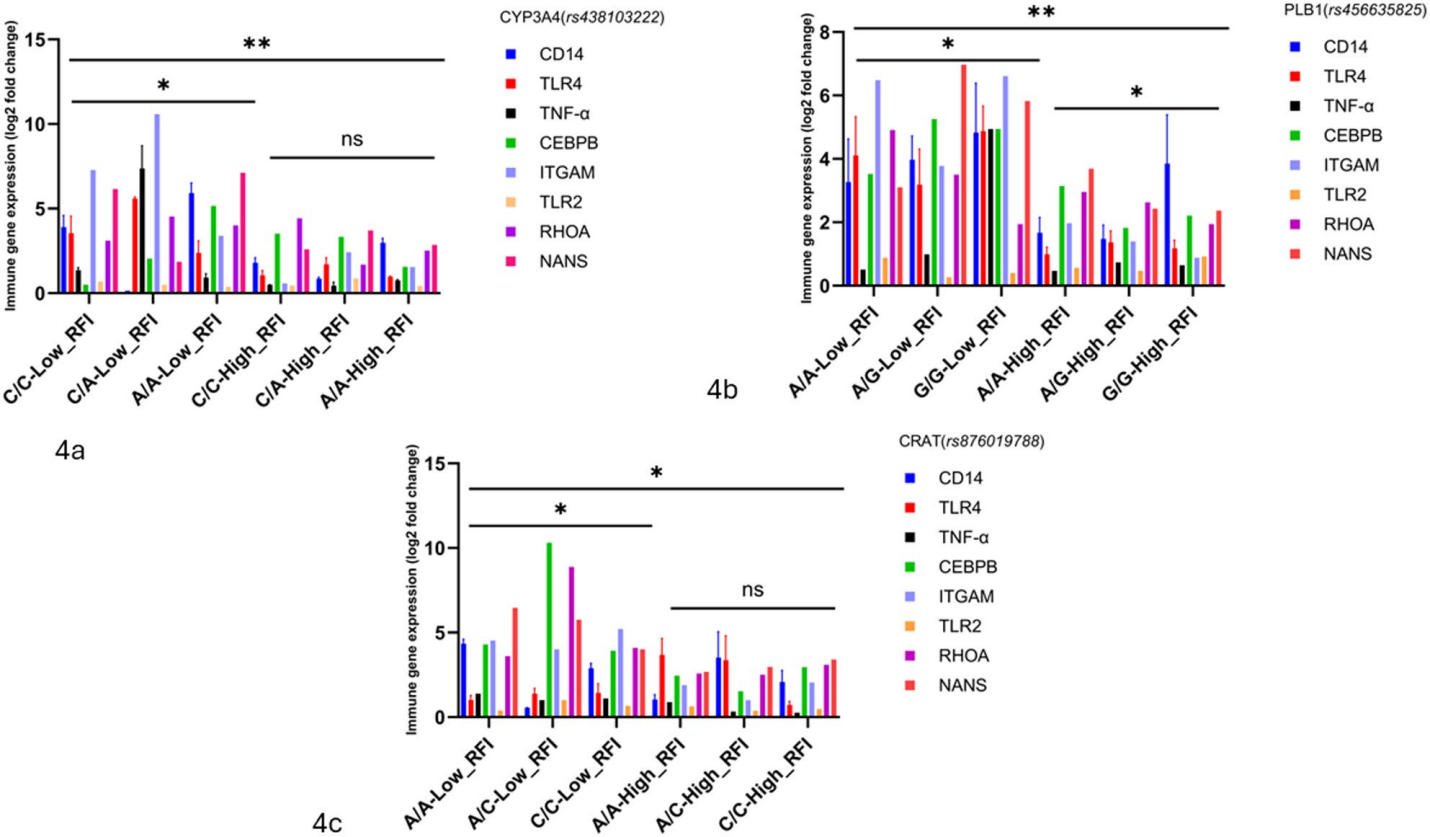
**a**-**c** Genotype-dependent immune gene expression in CYP3A4, PLB1, and CRAT variants. Asterisks denote statistical significance: *p* < 0.05 (*), ns = not significant. Low RFI = feed-efficient animals; High RFI = feed-inefficient animals

Furthermore, in Fig. 4a–c, the CYP3A4 rs438103222 A allele in low-RFI steers drives a 1.5–2.0-fold up-regulation of CD14, TLR4, TNF-α, CEBPB, and ITGAM (*p* < 0.05–0.01), evidencing an allele-specific amplification of innate immune signaling absent in high-RFI A-carriers; concurrently, PLB1 rs456635825 A/A homozygotes in low-RFI cattle not only augment PLB1 transcripts but also trans-activate TLR2, RHOA, and NANS by 1.4–1.8-fold (*p* < 0.05), linking phospholipid remodeling to cytoskeletal dynamics and sialic-acid–mediated immune pathways effects that are intermediate in G/A heterozygotes and abolished in high-RFI cohorts; by contrast, CRAT rs876019788 variants elicit no significant modulation of immune-gene expression in either RFI group (ns), underscoring its post-transcriptional regulatory role. Together, these findings designate CYP3A4 and PLB1 as bifunctional quantitative trait nucleotides that co-regulate metabolic detoxification and immune priming specifically in feed-efficient cattle.

### Allelic co-inheritance and haplotypic effects on RFI

Linkage disequilibrium (LD) was analyzed among three SNPs: CYP3A4 (rs438103222), PLB1 (rs456635825), and CRAT (rs876019788) (Table 7). LD values range from 0 to 1, with higher values indicating stronger correlations between loci. Statistical significance was determined using a predefined p-value threshold. LD analysis reveals a highly non-random allelic co-inheritance between CYP3A4(rs438103222) and PLB1(rs456635825) (D = 0.8981, *p* < 0.01), a moderate but significant haplotypic association between PLB1(rs456635825) and CRAT(rs876019788) (D = 0.2135, *p* < 0.05), and a minimal yet statistically significant disequilibrium between CYP3A4(rs438103222) and CRAT(rs876019788) (D = 0.0463, *p* < 0.05), collectively indicating non-random allelic segregation and suggesting putative epistatic interactions that could underlie feed-efficiency and immunometabolic quantitative trait loci.

**Table 7:** Linkage disequilibrium among CYP3A4, PLB1 and CRAT genetic polymorphisms

D-stat/*p*-values	CYP3A4(rs438103222)	PLB1 (rs456635825)	CRAT (rs876019788)
*CYP3A4(rs438103222)*	-	**	*
*PLB1 (rs456635825)*	0.8981	-	*
*CRAT (rs876019788*	0.0463	0.2135	-

*D-stat* Disequilibrium coefficient, *p*-values indicate statistical significance of allelic co-inheritance

*p* < 0.05 (*), *p* < 0.01 (**)

As shown in Table 8, haplotype reconstruction across the three loci generated eight discrete multi-locus allelic combinations (H1–H8), each defined by the phased variants at CYP3A4 (C/A), PLB1 (G/A), and CRAT (C/A) whose frequencies differ markedly between efficient (low‐RFI) and inefficient (high‐RFI) steers. The C-A-A haplotype (H1) is ubiquitous in low‐RFI cattle (42.5%) than in high‐RFI cattle (28.6%; *p* = 1 × 10⁻⁵), underscoring it as a protective architecture for efficient feed conversion. Conversely, the C-A-C haplotype (H3) is under‐represented in low‐RFI steers (7.5% vs. 13.5% in high‐RFI; OR = 0.50, *p* = 0.005), identifying it as a risk haplotype for inefficiency. Haplotypes H7 (A-A-A, 11.4% in high‐RFI only) and H8 (A-G-C, 3.5% in high‐RFI only) were exclusive to inefficient animals, further defining allelic combinations that predispose to poor feed utilization.

Other haplotypes (H2, H4, H5, H6) displayed intermediate frequencies with non-significant odds ratios, indicating neutral or context‐dependent effects. The clear divergence in H1 and H3 frequencies and the emergence of H7/H8 solely in the high‐RFI cohort reveals both additive and non‐additive (epistatic) interactions among CYP3A4, PLB1, and CRAT variants while reflecting a partial penetrance of the PLB1-G and CRAT-C alleles. These non-random allelic co-segregations (linkage disequilibrium D’>0.21 for significant pairs) reveal quantitative trait haplotypes that likely underpin immunometabolic resilience and feed-efficiency QTL. Such defined haplotype architectures provide high-resolution targets for marker-assisted selection aimed at optimizing bovine growth, feed conversion, and disease resistance.

**Table 8:** Haplotype frequencies of CYP3A4, PLB1, and CRAT in high- and low-RFI cattle

Haplotype	Haplotype definition			Haplotype frequency					
	***CYP3A4 (+ 328 C > A)***	***PLB1*** **(+ 1008** ***G > A*** **)**	***CRAT*** ***(+ 296 A > C)***	**High RFI**	**Low RFI**	**High RFI vs. Low RFI**		**OR (95% CI)**	***p*** **value**
H1	C	A	A	0.2862	0.425	0.3544	1.00		0.00001
H2	C	G	A	0.1888	0.325	0.2581	1.19 (0.16–8.93)		0.87
H3	C	A	C	0.1345	0.075	0.1062	0.50 (0.07–3.64)		0.005
H4	A	A	C	0.1155	0.05	0.0813	1.06 (0.14–7.95)		0.960
H5	A	G	A	0.0612	0.075	0.0669	1.21 (0.18–7.98)		0.85
H6	C	G	C	0.0655	0.05	0.0581	2.09 (0.27–6.22)		0.48
H7	A	A	A	0.1138	-	-	-		
H8	A	G	C	0.0345	-	-	-		

Significance: *p*-values indicate statistical significance of haplotype frequency differences. H1–H8 represent distinct multi-locus haplotypes defined by the phased alleles at three SNPs: CYP3A4 (C/A), PLB1 (G/A), and CRAT (C/A). Each haplotype reflects a unique combination of these variants and their frequency distribution between high-RFI and low-RFI cattle. These composite genetic architectures help identify favorable or unfavorable allele combinations associated with feed efficiency and immunometabolic resilience

*OR* Odds Ratio, *CI* Confidence Interval, *RFI* Residual Feed Intake, *Low RFI* feed-efficient animals, *High RFI* feed-inefficient animals

## Discussion

Our comprehensive analyses establish CYP3A4 and PLB1 as principal quantitative trait nucleotides (QTNs) modulating bovine feed efficiency, while the CRAT variant proves phenotypically inert. The allele-dosage–dependent performance gains particularly the superior feed conversion of CYP3A4 A/A homozygotes and the anabolic effect of the PLB1 A allele underscore the importance of single‐locus regulatory variation in shaping residual feed intake (RFI) phenotypes [7]. The deleterious heterozygote disadvantage at CYP3A4 further illuminates how mixed amino‐acid substitutions can disrupt enzyme kinetics, highlighting protein‐coding context as a critical consideration in QTN discovery [15, 28].

At the transcriptomic level, both CYP3A4 and PLB1 variants function as feed-efficiency-dependent cis‐eQTLs, driving robust up‐regulation of their own transcripts exclusively in low‐RFI cattle thus linking transcriptional control of xenobiotic metabolism and phospholipid remodeling to growth efficiency [29, 30]. These cis-effects were supported by significant genotype-expression associations as seen in our study, with expression levels stratified by genotype and RFI class [30]. Moreover, PLB1’s A allele exerts trans‐regulatory cross‐talk by amplifying CYP3A4 and CRAT expression and priming innate‐immune effectors, thereby integrating detoxification pathways with pathogen‐sensing circuits in efficient animals [19, 20, 31]. The absence of CRAT’s cis‐regulatory impact suggests its polymorphism operates primarily via post‐translational or enzymatic mechanisms within carnitine‐dependent energy pathways. However, the elevated ADFI observed for the A/C genotype should be interpreted cautiously. The relatively small A/C group and inherent sampling variability may have introduced outliers that contributed to the observed difference. Biological explanations of this locus require further functional validation. Future studies with larger genotype groups are needed to confirm whether this variant causally affects intake.

Linkage disequilibrium and haplotype reconstruction reveal a modular allelic architecture: strong LD between CYP3A4 and PLB1, moderate LD with CRAT, and two core low-RFI haplotypes that capture additive and epistatic synergies driving feed‐efficiency gains. This “supergene”‐like arrangement mirrors well‐characterized QTL clusters in livestock and underscores the value of multi‐locus haplotypes for explaining phenotypic variance beyond single SNPs [32–34]. Notably, the phased CYP3A4 A/A and PLB1 A haplotypes appear to coordinate xenobiotic metabolism, lipid remodeling, and immune activation, suggesting transcriptional synchrony and enzymatic compatibility across immunometabolic pathways. Such haplotype-driven regulatory synergy has been documented in livestock and model organisms, where co-inherited alleles enhance functional coherence and trait predictability [35–37].

The selection of CYP3A4, PLB1, and CRAT as candidate genes was supported by their mechanistic relevance to feed efficiency and immune function [7, 8]. Each encodes a metabolic enzyme with direct ties to nutrient processing and immune cell energetics, and the assayed SNPs represent non-synonymous variants with predicted functional consequences. Our data reveal that CYP3A4 and PLB1 polymorphisms not only associate with performance traits but also drive allele-specific transcriptomic changes in immune markers, reinforcing their role as bifunctional quantitative trait nucleotides. The observed linkage disequilibrium between CYP3A4 and PLB1 further supports a coordinated regulatory axis. While CRAT did not show strong phenotypic associations, its inclusion enabled a broader haplotypic analysis and highlighted the specificity of CYP3A4 and PLB1 effects. These findings validate our gene selection strategy and underscore the value of integrating functional annotation with expression profiling to identify high-impact markers for immunometabolic resilience.

Comparative genomics and regulatory pathway data further support the relevance of these loci. CYP3A4 is among the most polymorphic and inducible cytochrome P450 enzymes, with expression modulated by PXR and CAR in response to xenobiotics and dietary compounds [13, 14, 22]. PLB1 participates in lipid mediator synthesis and is upregulated during inflammatory responses, linking membrane remodeling to immune activation [19]. CRAT’s role in acetyl-CoA metabolism intersects with mitochondrial energy production and immune cell proliferation, although its transcriptional regulation appears less dynamic. These insights reinforce the functional coherence of our candidate gene set and highlight their utility for marker-assisted selection in beef cattle.

Translationally, these findings advocate for the integration of CYP3A4 and PLB1 QTNs, and their phased haplotypes into genomic selection frameworks. Incorporating such high-impact loci into genomic best linear unbiased prediction (GBLUP) or Bayesian models has been shown to enhance predictive accuracy by capturing both additive and non‐additive genetic variance [38, 39]. Future validation in diverse breeds and environments, coupled with multi-omics approaches (proteomics, metabolomics, chromatin conformation, and methylation profiling), will refine our understanding of the regulatory topology and fitness trade-offs underpinning feed efficiency and immunometabolic resilience [40, 41]. Ultimately, leveraging these immunometabolic QTNs and haplotypes in marker-assisted selection promises to accelerate genetic gain for productivity and animal health, forging more sustainable and resilient beef cattle production systems.

Although our cohort of 40 steers (20 high-RFI and 20 low-RFI) enabled robust genotype–phenotype comparisons, the limited sample size may constrain statistical power and elevate the risk of overfitting in logistic regression models. To address this, we implemented penalized regression and cross-validation procedures to reduce model complexity and improve generalizability. These methodological safeguards help ensure that the observed associations are biologically meaningful and not artifacts of sample size. Building on these findings, we note that while CRAT did not show strong phenotypic associations overall, unusually high variance among heterozygotes was observed. This pattern may reflect underlying biological complexity such as context-dependent enzyme activity or compensatory metabolic pathways, or potential genotyping artifacts. Further investigation using targeted resequencing, enzymatic assays, and expanded cohorts will be necessary to clarify the source and significance of this variance.

## Conclusion

In summary, our integrated analysis demonstrates that specific alleles of CYP3A4 (rs438103222) and PLB1 (rs456635825) constitute high-impact quantitative trait nucleotides that drive superior growth, feed conversion, and immunometabolic coordination in beef cattle, while the CRAT (rs876019788) variant lacks functional significance for feed‐efficiency traits. Through logistic regression, we showed that CYP3A4 A/A homozygotes and PLB1 A‐allele carriers achieve greater weight gains on reduced feed intake, and allele‐dosage effects at both loci manifest as feed‐efficiency–dependent cis‐ and trans‐regulatory enhancements of detoxification, lipid‐remodeling and innate‐immune pathways in low‐RFI animals. Concurrently, strong linkage disequilibrium between CYP3A4 and PLB1 and the identification of core low‐RFI haplotypes underscore a modular genetic architecture wherein additive and epistatic allelic synergies underpin phenotypic gains. These findings not only validate CYP3A4 and PLB1 as prime targets for marker‐assisted and genomic selection but also highlight the value of phased haplotypes in capturing complex variance. Moving forward, validating these loci across diverse breeds and environments alongside multi‐omics interrogation of post‐translational modifications, chromatin topology and metabolic flux will refine selection strategies and accelerate the sustainable genetic improvement of feed efficiency and animal health in commercial beef production.

## Data Availability

Data is provided within the manuscript.
